# Human Activity Determines the Presence of Integron-Associated and Antibiotic Resistance Genes in Southwestern British Columbia

**DOI:** 10.3389/fmicb.2018.00852

**Published:** 2018-05-01

**Authors:** Miguel I. Uyaguari-Díaz, Matthew A. Croxen, Zhiyao Luo, Kirby I. Cronin, Michael Chan, Waren N. Baticados, Matthew J. Nesbitt, Shaorong Li, Kristina M. Miller, Damion Dooley, William Hsiao, Judith L. Isaac-Renton, Patrick Tang, Natalie Prystajecky

**Affiliations:** ^1^Department of Pathology & Laboratory Medicine, The University of British Columbia, Vancouver, BC, Canada; ^2^BC Centre for Disease Control Public Health Laboratory, Vancouver, BC, Canada; ^3^Provincial Laboratory for Public Health, Edmonton, AB, Canada; ^4^Department of Laboratory Medicine and Pathology, Faculty of Medicine & Dentistry, University of Alberta, Edmonton, AB, Canada; ^5^Laboratory Services, Public Health Ontario, Toronto, ON, Canada; ^6^National Microbiology Laboratory, Public Health Agency of Canada, Winnipeg, MB, Canada; ^7^Coastal Genomics, Inc., Burnaby, BC, Canada; ^8^Pacific Biological Station, Nanaimo, BC, Canada; ^9^Department of Pathology, Sidra Medical and Research Center, Doha, Qatar

**Keywords:** antibiotic resistance genes, watersheds, metagenomics, high throughput screening, quantitative PCR, land-use, antibiotics

## Abstract

The dissemination of antibiotic resistant bacteria from anthropogenic sources into the environment poses an emerging public health threat. Antibiotic resistance genes (ARGs) and gene-capturing systems such as integron-associated integrase genes (*intI*) play a key role in alterations of microbial communities and the spread of antibiotic resistant bacteria into the environment. In order to assess the effect of anthropogenic activities on watersheds in southwestern British Columbia, the presence of putative antibiotic resistance and integrase genes was analyzed in the microbiome of agricultural, urban influenced, and protected watersheds. A metagenomics approach and high-throughput quantitative PCR (HT qPCR) were used to screen for elements of resistance including ARGs and *intI*. Metagenomic sequencing of bacterial genomic DNA was used to characterize the resistome of microbial communities present in watersheds over a 1-year period. There was a low prevalence of ARGs relative to the microbial population (<1%). Analysis of the metagenomic sequences detected a total of 60 elements of resistance including 46 ARGs, *intI1*, and groEL/*intI1* genes and 12 quaternary ammonium compounds (*qac*) resistance genes across all watershed locations. The relative abundance and richness of ARGs was found to be highest in agriculture impacted watersheds compared to urban and protected watersheds. A downstream transport pattern was observed in the impacted watersheds (urban and agricultural) during dry months. Similar to other reports, this study found a strong association between *intI1* and ARGs (e.g., *sul1*), an association which may be used as a proxy for anthropogenic activities. Chemical analysis of water samples for three major groups of antibiotics was below the detection limit. However, the high richness and gene copy numbers (GCNs) of ARGs in impacted sites suggest that the effects of effluents on microbial communities are occurring even at low concentrations of antimicrobials in the water column. Antibiotic resistance and integrase genes in a year-long metagenomic study showed that ARGs were driven mainly by environmental factors from anthropogenized sites in agriculture and urban watersheds. Environmental factors such as land-use and water quality parameters accounted for 45% of the variability observed in watershed locations.

## Introduction

Antibiotic resistance is recognized as a major emerging health threat worldwide. While not a new phenomenon, factors such as the global population growth, overuse, and limited development of novel antibiotics have increased overall the morbidity and mortality as well as the costs of treating of bacterial diseases (Spellberg et al., 2008; Colomer-Lluch et al., 2011).

Globally, the annual consumption of antibiotics is estimated to be 70 billion standard units/year for human use (Van Boeckel et al., 2014) and 63,151 ± 1,560 tons/year for livestock (Van Boeckel et al., 2015). Within the next 15 years, usage is predicted to increase by 30% and 67% for human and veterinary purposes, respectively (Gelbrand et al., 2015). Currently, mortality rates attributable to antimicrobial resistance are estimated to be 700,000 deaths annually (O’Neill, 2014). The same report suggests that this rate will increase to 10 million deaths/year by 2050; antimicrobial resistance will become a leading cause of death world-wide (O’Neill, 2014). While this number is being debated (de Kraker et al., 2016), an upward trend will undoubtedly continue as a consequence of the use and misuse of antibiotics.

It is estimated that between 75% and 90% of antibiotics are poorly absorbed by either humans or animal hosts and are excreted, unaltered, in feces or urine (Karthikeyan and Meyer, 2006; Sarmah et al., 2006; Bockelmann et al., 2009). Thus, spillage of antibiotics and their metabolites into the environment is widespread; contamination hotspots include hospital sewage discharges, health care facilities, and community wastewater treatment plants (WWTPs), pharmaceutical industry facilities, and confined animal feeding operations (Pruden et al., 2006; Berendonk et al., 2015). Terrestrial and aquatic ecosystems such as soil, rivers, streams, watersheds, groundwater, and sediments are the recipients of both antibiotic residues and antibiotic-resistant bacteria (Pruden et al., 2006; Baquero et al., 2008). While some antibiotics may degrade quickly, others accumulate in the soil or sediments and persist in the environment for a longer period of time (Shi et al., 2014; Xiong et al., 2015a). Surface water remains the main vehicle of dissemination of antibiotic resistant bacteria, antibiotic residues, and antibiotic resistance gene (ARG) elements into the environment (Heberer, 2002; Marti et al., 2014).

Microbial acquisition of ARGs occurs through a variety of gene transfer systems using genetic elements such as conjugative plasmids, transposons, and integrons (Bennett, 2008; Garriss et al., 2009). Moreover, horizontal gene transfer enables genes to move from one bacterial cell to another and from one ecosystem to another (Bennett, 2008). The role of elements such as phages and integrons in the spread of resistance appears to be significant in freshwater ecosystems (Lupo et al., 2012) and has been well documented using culture-independent approaches (Marti et al., 2013; Stalder et al., 2014; Xiong et al., 2014, 2015b). In particular, integrons are considered the main agents of bacterial evolution due to their role in the dissemination of ARGs, development of multiple drug resistance, and their ability to add gene structures into bacterial genomes (Mazel, 2006; Joss et al., 2009; Cambray et al., 2010; Gillings, 2014). These assembly platforms containing gene cassettes with a variety of functions, mainly antimicrobial resistance, are composed of a gene encoding integrase (*intI*), a recombination site (*attI*), and a promoter (P_C_) located upstream of the gene cassette (Fluit and Schmitz, 2004; Gillings, 2014). Among these elements, *intI* genes are considered the signatures to differentiate integron classes (Sá et al., 2010). In this context, there are at least five *intI* classes (Ravi et al., 2014); the first three classes of *intI* (*intI1*, *intI2*, and *intI3*) are mainly linked with horizontal gene transfer among multiple bacterial species (Shibata et al., 2003; Fluit and Schmitz, 2004; Ramirez et al., 2005; Xu et al., 2007; Gillings et al., 2008, 2015; Wright et al., 2008; Partridge et al., 2009; Rodriguez-Minguela et al., 2009; Barkovskii et al., 2010; Ravi et al., 2014), while the other two classes, *intI4* and *intI5*, are chromosomal integrases harbored by *Vibrio* isolates conferring resistance to trimethoprim (Diaz-Mejia et al., 2008; Ravi et al., 2014). It is hypothesized that ancestral integrons may have not harbored multiple ARGs compared to modern integrons, which encode resistance to quaternary ammonium compounds/disinfectants, sulfonamides, and aminoglycosides and exemplify the increase in antibiotic usage (Gillings et al., 2009; Ravi et al., 2014; Adesoji et al., 2017) in a wide variety of environments.

Several metagenomic studies have been conducted in aquatic ecosystems analyzing the resistome of microbial communities from sediments, WWTPs, and effluents (Uyaguari et al., 2011; Gomez-Alvarez et al., 2012; Zhu et al., 2013; Baumlisberger et al., 2015). Only a few of these studies, however, have focused on ARGs or elements of resistance in surface water or watersheds (Li B. et al., 2015; Staley et al., 2015). Since watersheds are a key source of drinking water, they may represent a pivotal role in the spread of antibiotic resistance. As watersheds are impacted by land use and anthropogenic activities, the water they provide flows into other larger aquatic ecosystems such as rivers, lakes, basins, estuaries, and salt water bodies. In the present study, we collected raw surface water samples from three watersheds in southwestern British Columbia, Canada, over a 1-year period. Each watershed was characterized by different land use (agricultural, urban, and protected). Metagenomic sequencing of the bacterial fraction and high-throughput quantitative PCR were used to characterize and quantify elements of resistance in study water samples. Understanding the interplay between land use, watersheds, and the dissemination of ARGs or genetic elements associated to resistance, is important to understanding and planning to prevent future impacts to both the public health and the environment.

## Materials and Methods

**FIGURE 1:**
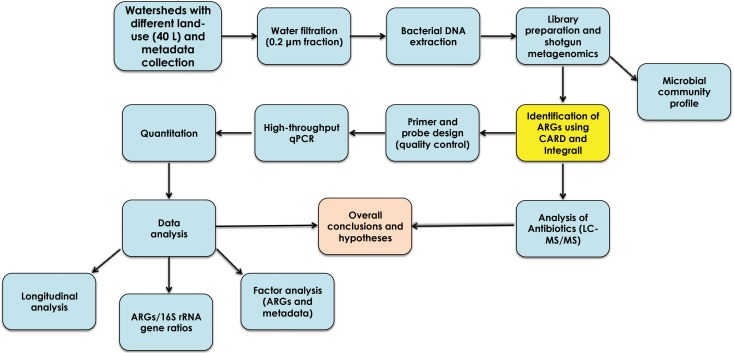
Experimental design used in the characterization and quantitation of antibiotic resistance genes from agriculture, urban, and protected watersheds in southwestern British Columbia.

In the present study, we combined shotgun metagenomics and high-throughput quantitative PCR (HT qPCR) to detect, characterize, and quantify ARGs and elements of resistance in watersheds with different land use. **Figure 1** summarizes the experimental design used in the study.

### Sample Collection

A case-control design was used to characterize spatial and temporal distribution of representative antibiotic resistance and integrase genes in watersheds. Each sampling study site represented different land-use: agriculture (Agricultural Upstream or AUP site located in a forested area with minimal housing, Agricultural Polluted or APL site receiving effluents from multiple farms, and Agricultural Downstream or ADS site, 2.5 km from APL and fed by waters from APL and another tributary with both agricultural and some urban influence); urban (Urban Polluted or UPL site characterized by residential development in a mountainous forest area, and urban downstream or UDS site, 1 km from UPL and running through residential, forested, and park areas); and non-impacted (Protected Upstream or PUP site, in a protected watershed that provides drinking water for a large community, and Protected Downstream or PDS site, 16 km from PUP). This last site was located after the drinking water supply (raw reservoir water) passed through a 9-km pipe (2 m in diameter; **Supplementary Figure S1**). Agricultural, urban and protected watersheds are not connected; they were separated by a distance of at least 63 km. A total of 89 samples were collected during a 13-month period (April 2012 to April 2013). Samples related to urban watersheds were obtained in a 12-month period. All 40-l samples were collected in sterile plastic carboys. Samples were pre-filtered *in situ* using a 105-μm spectra/mesh polypropylene filter (SpectrumLabs, Rancho Dominguez, CA, United States) to remove larger debris. The filtrate was kept on ice, and transported to the British Columbia Centre for Disease Control Public Health Laboratory (BCCDC PHL) for processing. All samples were kept at 4°C until analysis. An additional 250-ml subsample for each of the 89 samples was collected in amber high-density polyethylene sterile bottles without prefiltration, transported to the laboratory and stored at -80°C for further analysis for antibiotics.

### Metadata

Water quality parameters were measured *in situ* using a YSI Professional Plus handheld multiparameter instrument (YSI Inc., Yellow Springs, OH, United States). Physico-chemical parameters of watersheds included: temperature (°C), dissolved oxygen (mg/l), specific conductivity (μS/Cm), total dissolved solids (mg/l), salinity (PSU), pressure (mmHg), and pH. Turbidity (NTU) was measured using a VWR turbidity meter model No. 66120-200 (VWR, Radnor, PA, United States). Water flow data (m^3^/s) was determined *in situ* using a Swoffer 3000 current meter (Swoffer Instruments, Seattle, WA, United States). Total coliform and *Escherichia coli* counts were determined using the Colilert-24 testing procedure (IDEXX Laboratories, Westbrook, ME, United States). Laboratory analysis included dissolved chloride (mg/l) and Ammonia (mg/l) using automated colorimetry (SM-4500-Cl-G) and spectrophotometry (SM-4500NH3G). Other nutrients such as chlorophyll *a* (Welschmeyer, 1994), orthophosphate (Murphy and Riley, 1962), nitrite, and nitrate (Wood et al., 1967) were also analyzed.

### Filtration and DNA Extraction

The bacterial fraction of each water sample was captured by passing water first through a 1 μm-Envirochek HV sampling capsules (Pall Corporation, Ann Harbor, MI, United States), followed by further filtration using a 0.22-μm 142 mm Supor-200 membrane disc filters (Pall Corporation, Ann Harbor, MI, United States) that retained bacterial cells as previously described (Uyaguari-Diaz et al., 2015, 2016). Filters were cut into small strips (1 cm × 1 cm) using sterile scissors and placed into 50 ml sterile centrifuge tubes (VWR, Radnor, PA, United States). Bacterial-sized cells captured on the Supor-200 membrane disc filters were then washed with 15 ml of 1× phosphate buffered saline (PBS) and 0.01% Tween (pH 7.4). Mechanical procedures involving vigorous vortexing for 20 min in 50 ml tubes vortex adaptors and centrifugation (3,300 × *g*, 15 min at 4°C) were used to remove and further concentrate cells. The supernatant was removed up to the 5 ml mark, and then bacterial cells were resuspended and aliquots of 1 ml distributed into sterile 1.7 ml microcentrifuge tubes. Bacterial cells were further pelleted down at 10,000 × *g*, 10 min at 4°C, supernatant was removed up to ∼200 μl mark. A total of five cell aliquots per sample were then stored at -80°C for DNA extraction. DNA was extracted from multiple concentrated cell aliquots using the PowerLyzer PowerSoil DNA kit (MoBio, Carlsbad, CA, United States) by following the manufacturer’s instructions. DNA was precipitated using 10% 3M sodium acetate and 2× 100% ethanol, and 5 μl of 5 μg/μl linear acrylamide, washed with 1 ml of 70% ice-cold ethanol, and eluted in 34 μl of 10 mM Tris solution. DNA concentration and purity was assessed with Qubit fluorometer (Life Technologies, Carlsbad, CA, United States) and NanoDrop spectrophotometer (NanoDrop technologies, Inc., Wilmington, DE, United States), respectively.

### Metagenomic Sequencing

To characterize microbial communities and subsequently identify antibiotic resistance elements in samples, libraries were prepared using Nextera XT DNA Sample Preparation kit (Illumina, Inc., San Diego, CA, United States). Sequencing libraries were generated using 1 ng of DNA, according to the manufacturer’s instructions, with a gel size selection modification using Coastal Genomics’ Ranger Technology (Uyaguari-Diaz et al., 2015). Metagenomic sequencing was performed on a MiSeq platform (Illumina, Inc., San Diego, CA, United States) using an Illumina MiSeq V2 2 × 250 bp paired-end reagent kit. Seventeen water samples were multiplexed on each MiSeq cartridge. To balance the diversity and account for batch effect in each sequence run, we included five samples from agricultural, five samples from urban, five samples from protected watersheds, one negative or background control, and one mock community (Peabody et al., 2015). Genomic DNA from known bacterial species were pooled in equal molar amounts and processed like indicated in the Nextera XT DNA sample preparation kit (Illumina, Inc., San Diego, CA, United States). Raw bacterial reads were created and are available as part of a large-scale Watershed Metagenomics project, BioProject ID: 287840^1^.

### Bioinformatics Workflow

Trimmomatic version 0.32 (Bolger et al., 2014) was used to remove adapters using the sequences packaged with the A5-Miseq assembly pipeline (Coil et al., 2015). Low quality and short sequences (<75 nt) were discarded. Sequences were then assembled using PandaSeq (Masella et al., 2012); unassembled pairs were retained. *De novo* assembly was conducted on the assembled and unassembled pairs using MEGAHIT (Li D. et al., 2015) and contigs shorter than 200 nt were discarded. Nucleotide sequences were aligned against the comprehensive antibiotic resistance database (CARD; McArthur et al., 2013) and Integrall (Moura et al., 2009) using BLAST (Altschul et al., 1990).

### Probe and Primer Design

Fully annotated genes from CARD encoding resistance to known antibiotics were used to design primers and probes for qPCR using Primer3Plus software (Untergasser et al., 2007). Specific primers for *intI1-3* genes were selected from the literature (Barraud et al., 2010). The primers were first checked for sensitivity and specificity by SYBR green-based PCR (data not shown). TaqMan probes (Life Technologies, Carlsbad, CA, United States) were then designed and validated for the primers to provide suitable sensitivity and specificity (data not shown). Primers with low sensitivity and specificity were excluded from the HT qPCR. Assays with cycle threshold (Ct) values greater than 35 were not included in the HT qPCR run using the BioMark system (Fluidigm Corporation, South San Francisco, CA, United States). **Table 1** summarizes primers and probes used in this study. All probes used a 5′ 6-FAM dye with an internal ZEN quencher and 3′ Iowa Black fluorescent quencher (Life Technologies, Carlsbad, CA, United States).

**Table 1:** Primers and probes used in quantitative PCR of antibiotic resistance gene fragments.

Gene	Mechanism	Forward primer (5′ → 3′)	Reverse primer (5′ → 3′)	(6-FAM) Probe/ZEN (IABkFQ) (5′ →3′)	Fragment size (bp)
*aacA1*	Encodes aminoglycoside 6′-N-acetyltransferase.	GTAGCGGTGACCATCGAAAT	TACGGCCACAGTAACCAACA	GGTGCTAAGCGTCATTGAGC	162
*aada1*	Encodes aminoglycoside-3′-(9)-O-adenylyltransferase.	TGATTTGCTGGTTACGGTGA	AGTTCGCGCTTAGCTGGATA	ATTGTTGTGCACGACGACAT	177
*bla(IMP)*	Encodes metallo beta-lactamase.	AGTGAAATTGGGAACGCATC	CAAACCATAAAACCGCGACT	TAACAAGTCGTTGCAGCACC	169
*strA*	Encodes streptomycin phosphotransferase.	TGACTGGTTGCCTGTCAGAG	AATTGCCGTTATCACCAAGC	TTTGTTTTTCGACGTGGTGA	217
*strB*	Encodes streptomycin phosphotransferase, StrB.	ATGATGCAGATCGCCATGTA	GAAATTGCAGCGGAACTGAT	TTTGATCGGCTATAATCGCC	222
*sul1*	Encodes dihydropteroate synthase.	AGGCTGGTGGTTATGCACTC	CCGACTTCAGCTTTTGAAGG	ACGAGATTGTGCGGTTCTTC	238
*sul2*	Encodes sulfonamide-resistant dihydropteroate synthase, Sul2.	GCGGGTTGATAACTGTCGAG	TTTCGGCATCGTCAACATAA	GACCGAGGTCGATCACATCT	249
*tet(32)*	Encodes a Tetracycline resistance protein Tet(32).	ATGGAGGGGGTTCTTTATGG	GAGATATTCCTGCGGTGCAT	CCTATCGTATTGGAGCAGGC	222
*tet(Q)*	Encodes a Tetracycline resistance protein Tet(Q).	CCAGACAGGTCCGAAGAGAG	TGGACCTTTACGGAAAATCG	TTGAAGACCCGTCTTTGTCC	182
*tet(W)*	Encodes a Tetracycline resistance protein Tet(W).	GGTGCAGTTGGAGGTTGTTT	TCAAGTATCCCAGCGAAACC	AAAGGAACCCTCCGTCATTT	230
*IntI1*^∗^	Integron-encoded integrase class 1	GCCTTGATGTTACCCGAGAG	GATCGGTCGAATGCGTGT	ATTCCTGGCCGTGGTTCTG GGTTTT	196
*IntI2*^∗^	Integron-encoded integrase class 2	TGCTTTTCCCACCCTTACC	GACGGCTACCCTCTGTTATCTC	TGGATACTCGCAACCAAGTTA TTTTTACGCTG	195
*IntI3*^∗^	Integron-encoded integrase class 3	GCCACCACTTGTTTGAGGA	GGATGTCTGTGCCTGCTTG	CGCCACTCATTCGCCACCCA	138
*16S rRNA*^∗∗^	Ribosomal RNA gene	ATGGYTGTCGTCAGCT	ACGGGCGGTGTGTAC	CAACGAGCGCAACCC	352


*^∗^Barraud et al., 2010; ^∗∗^Ritalahti et al., 2006.*

### Standard Curves

Genomic DNA from either agricultural or urban impacted watersheds was used as template to generate amplicons for each ARG (**Table 1**). Non-template controls used nuclease free-water (Promega Corporation, Fitchburg, WI, United States). DNA from purified strains of *E. coli* ATCC 25922, and *E. coli* strains JVC1076, JVC1170, and JM109 (generous gift from Drs. Davies, Miao, and Villanueva, The University of British Columbia) were used as amplification controls for 16S rRNA gene, *intI1*, *intI2*, and *intI3* genes, respectively. PCR conditions were defined as follows: 94°C for 5 min, followed by 35 cycles of 94°C for 30 s, 55°C (50°C were used for *intI* genes, **Table 1**) for 45 s, 72°C for 1 min, and a final extension at 72°C for 10 min. Amplicons were visualized on a 1.5% agarose gel. PCR amplicons were purified with a QIAQuick PCR Purification Kit (Qiagen Sciences, Inc., Germantown, MD, United States) according to the manufacturer’s instructions. The purified amplicons were ligated into pCR2.1-TOPO cloning vectors (Invitrogen, Carlsbad, CA, United States) and transformed into One Shot *E. coli* DH5 α-T1^R^ competent cells following the manufacturer’s protocol. Transformants were grown overnight at 37°C in lysogeny broth with 50 μg/ml of kanamycin. Plasmids were extracted and purified using PureLink Quick Plasmid Miniprep Kit (Life Technologies, Carlsbad, CA, United States), and quantified using a Qubit dsDNA high sensitivity kit on a Qubit 3.0 fluorometer (Life Technologies, Carlsbad, CA, United States). Plasmids for each ARG and integrase gene class were end-sequenced using an ABI 3130xl Genetic Analyzer (Life Technologies, Carlsbad, CA, United States) with M13 forward primer (-20) (5′-GTAAAACGACGGCCAG-3′) and M13 reverse primer (5′- CAGGAAACAGCTATGACC-3′) using BigDye Terminator version 3.1 cycle sequencing kit (Applied Biosystems, Warrington, United Kingdom). The resultant set of DNA sequences for each ARG and integrase classes were searched against the GenBank database using blastx with default settings.

Plasmid DNA harboring amplicon gene standards were linearized by digestion with the BamHI endonuclease (Life Technologies, Carlsbad, CA, United States). Serial dilutions (*n* = 6) of the linearized plasmids were multiplexed and used as templates to generate standard curves. Additional bioinformatic analysis screened for potential primer collision cases against the BLAST nt database using up to 1000 nt as maximum collision distance (**Supplementary Spreadsheet File S1**). Estimates of bacteria, antibiotic resistance, and integrase gene were determined using 16S rRNA, ARGs and *intI* gene fragments, respectively (**Table 1**). GCNs per ml of sample were calculated as previously described (Ritalahti et al., 2006).

### High-Throughput Multiplex Quantitative Polymerase Chain Reaction

DNA extracts from watershed samples were diluted 10-fold and 1.25 μl of DNA from each sample was pre-amplified with low-concentration primer pairs (0.2 μM) corresponding to all assays (**Table 1**) in a 5 μl reaction volume using TaqMan Preamp Master Mix (Life Technologies, Carlsbad, CA, United States) according to the BioMark protocol (Fluidigm Corporation, South San Francisco, CA, United States). Unincorporated primers were removed using ExoSAP-IT High-Throughput PCR Product Clean Up (MJS BioLynx Inc., Brockville, ON, Canada) and samples were diluted 1:5 in DNA Suspension Buffer (TEKnova, Hollister, CA, United States).

The pre-amplified products were run on the BioMark system (Fluidigm Corporation, South San Francisco, CA, United States) using 96.96 dynamic arrays. Five μl of 10× assay mix (9 μM primers and 2 μM probes) were loaded to the assay inlet, while 5 μl of sample mix (2× TaqMan Mastermix; Life Technologies, Carlsbad, CA, United States), 20× GE Sample Loading Reagent, nuclease-free water, and 2.25 μl of pre-amplified DNA were loaded to each sample inlet of the array following the manufacturer’s recommendations. After mixing the assays and samples into the chip with an IFC controller HX (Fluidigm Corporation, South San Francisco, CA, United States), qPCR was performed with the following conditions: 50°C for 2 min, 95°C for 10 min, followed by 40 cycles of 95°C for 15 s and 60°C for 1 min. Samples were run in quadruplicates for all six standards, for the 89 environmental samples and for the one no-template control.

### Analysis of Antibiotic Residues

A subset of water samples (*n* = 19) stored at -80°C was sent to a commercial laboratory to screen for antibiotic residues. This included 13 water samples from APL and one sample from the other sites (AUP, ADS, UPL, UDS, PUP, and PDS). Selection of antibiotic residues for testing was based on the major groups of ARGs found through the CARD database and included: ampicillin (β-lactam group); sulfamethoxazole (sulfonamide group); and chlortetracycline, doxycycline, oxytetracycline, and tetracycline (tetracycline group). Residues of ampicillin and sulfamethoxazole were analyzed by LC/MS following the methods described in EPA 549.2 (EPA, 1997), while the tetracycline group was tested by LC/MS–MS following a modified EPA 549.2 method (EPA, 1997).

### Data Analysis

To estimate the percentages of ARGs and *intI*, we used the number of contigs matching CARD and Integrall divided by the total number of contigs per site (**Table 2** and **Supplementary Spreadsheet File S2**). In order to estimate the “true” resistance potential in the microbial community, a manual curation was conducted on these matching hits. Mechanisms such as mutation based resistance genes, target proteins for resistance genes, and regulators for resistance genes were not included in downstream analysis. GCNs and metadata were transformed using log_10_ function for analysis. Longitudinal analysis (PROC mixed with repeated measures) was conducted on the qPCR data for ARGs and *intI* to detect differences between watershed sites over time. Replicate values of all antibiotic resistance and integrase genes for each site were averaged and these values were introduced into the model. Tukey’s test was used to determine time effect among the different sites. Spearman’s rank correlation analysis was also conducted among ARGs, *intI* and water quality parameters. Factor analysis employed a parsimax oblique rotation that included metadata and the ratio of each ARG normalized by the 16S rRNA gene (as estimated by HT qPCR platform) measured in watershed locations. Statistical analyses were performed using Statistical Analysis System (SAS, version 9.4 for Windows). A *p*-value of 0.05 was assumed for all tests as a minimum level of significance. Additional taxonomic characterization was conducted with Taxonomer (Flygare et al., 2016) and microbial community diversity and richness indices were calculated using PAST (version 3.18; Hammer et al., 2001).

**Table 2:** Year-long summary statistics of bacterial metagenomes in watershed locations and relative abundance (expressed as %) of antibiotic resistance genes using 2 databases: CARD and Integrall.

Watershed locations	Raw reads	Contigs	Read length (Post QC)	GC content (Post QC)	CARD	Integrall
AUP	23406732	372969	367 ± 56	58 ± 3	0.48 ± 0.19	0.25 ± 0.12
APL	19677726	662111	402 ± 43	53 ± 3	0.48 ± 0.29	0.84 ± 2.72
ADS	21785092	822639	399 ± 43	52 ± 2	0.43 ± 0.19	0.21 ± 0.37
UPL	18725664	305303	369 ± 70	56 ± 3	0.43 ± 0.16	0.21 ± 0.13
UDS	20169636	263575	333 ± 32	56 ± 2	0.45 ± 0.12	0.21 ± 0.09
PUP	21438298	226701	322 ± 23	57 ± 3	0.38 ± 0.13	0.22 ± 0.10
PDS	24555028	1297188	427 ± 33	53 ± 2	0.14 ± 0.02	0.02 ± 0.03


*Values represent the average percentage of detected genes (percentage of total contigs in which genes of interest were detected) ± standard deviation for each sampling location).*

## Results and Discussion

Eighty-nine surface water samples among seven sampling sites located in three watersheds of differing land-use in southwestern British Columbia were analyzed. Samples were collected at regular intervals over the course of one year. Bacterial metagenomic sequencing generated 36 Gb for this dataset. CARD and Integrall were selected over other databases due to their characteristics of available ARG spectrum data and resources including functionality and features (Xavier et al., 2016). A total of 3.95 million contigs (≥200 bp) out of 150 million reads were assembled and analyzed using CARD and Integrall.

From the total sequencing reads identified by the databases (CARD and Integrall) as elements of resistance (**Table 2**), we observed a large proportion (∼96%) of them as housekeeping function-associated genes such as elongation factors (55.4%), DNA-directed RNA polymerase (23.8%), DNA topoisomerases/gyrases (10.6%), resistance nodulation cell division (3.2%), and transport systems (2.0%) (**Supplementary Figure S2B**). Although these functions can be targeted by antibiotics such as elfamycin, daptomycin/rifampin, and fluoroquinolones, ethambutol, or other drugs (**Supplementary Figure S2A**), identification of these genes is not in itself an indication of resistance; the universality of these structural genes as cellular protection mechanisms or regulators has been widely documented in a variety of environments (Yu and Zhang, 2012; Ma et al., 2014; Baumlisberger et al., 2015; Chen et al., 2015). Thus, we further manually curated both databases and found that the remaining percentage (∼4%) of these genes encoded resistance to at least one antibiotic. We also included tetracycline resistance genes as this antibiotic group acquires new genes through mobile plasmids or transposons (Agerso and Sandvang, 2005; Roberts and Schwarz, 2009). After the curation process, using CARD, we identified a total of 12,044 hits from 353 contigs as elements of resistance, with grouping into 46 ARG types (conferring resistance to at least one antibiotic). Using Integrall, we observed a total of 6,688 hits with 1200 contigs mostly associated to *intI1* and 12 *qac* genes. These contigs per watershed location, time point, and database (CARD and Integrall) are provided in **Supplementary Spreadsheet File S2**. The results from this study describe only genes with resistance potential within microbial communities in watersheds.

### Main Classes of Antibiotics and Mechanisms of Antibiotic Resistance

**FIGURE 2:**
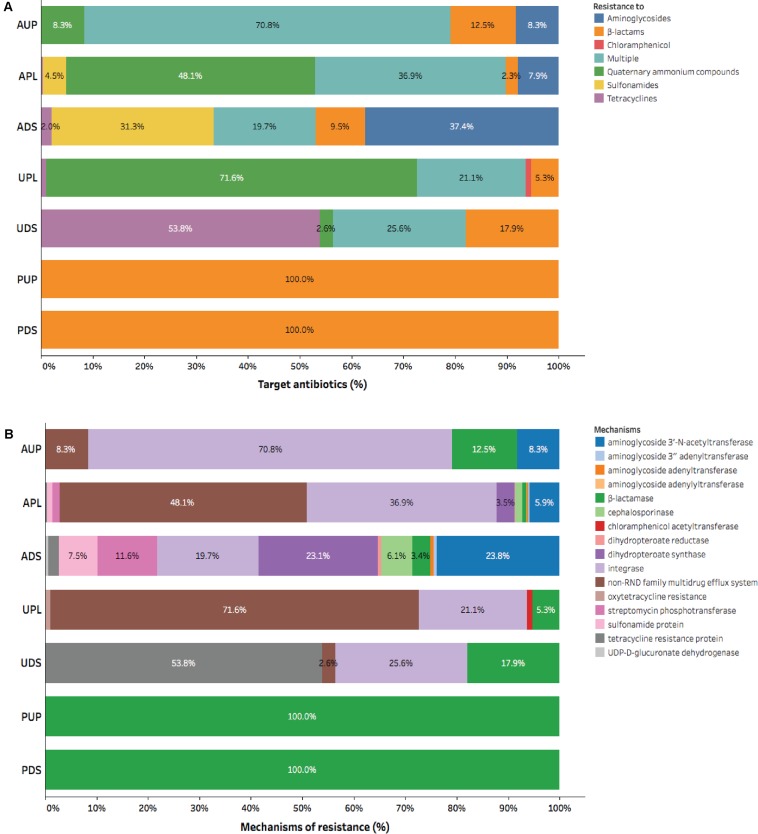
Composition of antibiotic resistance gene categories assigned by CARD in watershed locations: **(A)** Group of antibiotic, and **(B)** Mechanism of action. AUP, agricultural upstream site; APL, agricultural polluted; ADS, agricultural downstream; UPL, urban polluted; UDS, urban downstream; PUP, protected upstream; PDS, protected downstream.

A more diverse group of elements of resistance conferring resistant to aminoglycosides, β-lactams, sulfonamides, tetracyclines, and multiple antibiotics was observed in agriculture impacted sites (APL and ADS) compared to AUP, urban, and protected watersheds (**Figure 2**). Aminoglycoside resistance genes were only detected in agricultural influenced watersheds and ranged from 7.9% (APL) up to 37.4% (UDS) (**Figure 2A**). Aminoglycoside acetyltransferases and dihydropteroate synthase were the most common mechanisms conferring resistance to aminoglycosides. They were found in agricultural impacted sites with average values of 14.9% and 13.3%, respectively (**Figure 2B**). Other aminoglycoside resistance mechanism present in agricultural impacted watersheds included streptomycin phosphotransferase (APL: 1.4% and ADS: 11.6%; **Figure 2B**). Genes involved in resistance to β-lactam antibiotics made up at least 2.3% of ARGs in the APL and were detected in all sites (**Figure 2A**). Although β-lactamases were present in all watersheds, cephalosporinases were only observed in APL and ADS with values of 1.4% and 6.1%, respectively. In the same context, genes conferring resistance to sulfonamides were only observed in APL and ADS with values of 4.5% and 31.3%, respectively. Moreover, relative abundance of tetracycline resistance genes had values of 0.4% in APL and 2.0% in ADS. Interestingly, multiple resistance as indicated by the integron-integrase genes class 1 were present in agricultural and urban influenced watersheds with average values of 42.5% and 23.4%, respectively. Furthermore, *qac* genes were observed in higher relative abundance in impacted watersheds (APL: 48.1% and UPL: 71.6%).

In urban impacted watersheds a higher relative abundance of tetracycline resistance genes was observed compared to other watersheds. In this context, UDS had a higher relative abundance (53.8%) of tetracycline resistance genes compared to other watershed locations. Furthermore, a resistance mechanism associated to chloramphenicol acetyltransferase (1.1%; **Figure 2B**) could be only detected in UPL. Finally, using metagenomics we could only identify ARGs to β-lactams in protected watershed sites (**Figure 2**). The ubiquity of β-lactams in protected watersheds may reflect microbial communities unique to this soil/sediment as previously demonstrated in low impact or undisturbed environments (Allen et al., 2009; Agga et al., 2015) or may reflect biofilm formation (Kostakioti et al., 2013; Balcazar et al., 2015) within the pipe carrying water from a drinking water catchment to the community (samples were collected that flowed out of this pipe). Overall, our results confirmed the prevalence of antibiotic resistance determinants to these major classes of antibiotics (β-lactams, aminoglycosides, sulfonamides, and tetracyclines) and elements of resistance such as *intI1* and *qac* genes in anthropogenic-influenced environments (Szczepanowski et al., 2009; Zhu et al., 2013; Wichmann et al., 2014; Baumlisberger et al., 2015). Gene richness and phylogenetic information associated with these mobile genetic elements are described below.

### Diversity and Distribution of Antibiotic Resistance Genes

**FIGURE 3:**
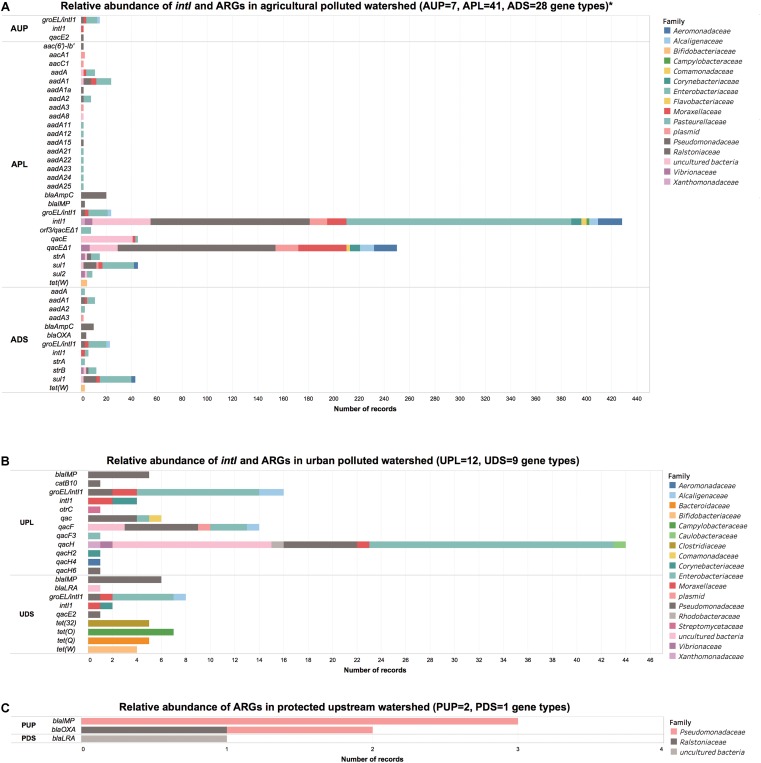
Number of records of integrase and antibiotic resistance genes in **(A)** agricultural, **(B)** urban DNA, and **(C)** protected watershed locations. AUP, agricultural upstream site; APL, agricultural polluted; ADS, agricultural downstream; UPL, urban polluted; UDS, urban downstream; PUP, protected upstream; PDS, protected downstream. ^∗^Single records have been filtered in agricultural watershed location for visualization purposes. For further details refer to Supplementary **Supplementary Spreadsheet File S2**.

From the bacterial DNA metagenomes using CARD and Integrall, we identified a total of 46 ARGs, *intI1*, and groEL/*intI1* genes and 12 *qac* genes associated with the different watersheds distributed in 28 bacterial families. **Figure 3** shows the distribution of ARGs and elements of resistance in the watershed locations with the phylogenetic affiliation at family level based on original input from the databases.

Forty-seven ARGs including *intI1* and groEL/*intI1* genes and five *qac* genes were observed in agriculture-influenced watersheds and distributed among 24 bacterial families. A large number of these ARGs and elements of resistance were observed in APL (*n* = 41) and ADS (*n* = 28), while less richness of ARGs genes was found in AUP (*n* = 7; **Figure 3A**). In AUP, *intI1* were observed in 71% of the ARGs, with 53% associated to members of the *Pseudomonadaceae* family. A small number of genes conferring resistance to β-lactams (*n* = 3) and aminoglycosides (2) were observed distributed among *Pseudomonadaceae*, *Enterobacteriaceae*, *Caulobacteraceae*, and *Ralstoniaceae* families. Moreover, *qac* genes in AUP were observed only in members of the family *Pseudomonadaceae* (8.3%).

In APL, *intI1* genes represented a large proportion of the elements of resistance (48%) and were distributed mainly between *Enterobacteriaceae* (42.1%), *Pseudomonadaceae* (27.5%), uncultured bacteria (10%), *Aeromonadaceae* (4.1%), *Moraxacellaceae* (3.3%), and plasmids (3.1%), (**Figure 3A**). Other representative ARGs in APL included *aadA* genes (15.3%), mainly *aadA1* with 8.1%, *sul1* (10.0%), *ampC* (4.4%), *aac* (2.2%), and *strA* and *sul2* with 2.0% each. In ADS, *sul1* (30.6%) distributed mostly between *Enterobacteriaceae* (55.5%) and *Pseudomonadaceae* (22.2%), was the ARG with the highest relative abundance followed by *intI1* (19.7%). Other abundant genes in ADS included *strB* (9.5%) and *aadA1* (8.8%) with the family *Enterobacteriaceae* representing 44.4% of these mobile genetic elements. Although detected in low relative ratios, *tet(W)* was found in agriculture impacted watersheds (APL and ADS) with average values of 1.3% and affiliated to members of the family *Bifidobacteriaceae*. Overall, we observed a wide range of ARGs in both agriculture-impacted sites, APL (*n* = 41) and ADS (*n* = 28) (**Figure 3A**). The relative higher abundance of genes such as *intI1* and presence of *sul1* in APL and ADS is consistent with previous studies reporting the prevalence of these genes in agriculture impacted environments (Byrne-Bailey et al., 2009; Gaze et al., 2011; Stalder et al., 2012; Wang N. et al., 2014; Gillings et al., 2015).

Urban sites were less diverse in ARGs compared to APL and ADS. In UPL and UDS, a total of 12 and 9 ARGs were observed, respectively. From the sequencing data, we observed that urban sites shared at least 2 ARG types between them (*intI1* and *bla*_IMP_ genes). Similar to the agriculture impacted sites, UPL and UDS had high relative abundances of *intI1* genes (∼23.4%), which may be an indication of the prevalence of these genes in anthropogenic impacted environments (Stokes and Gillings, 2011; Stalder et al., 2012, 2014). In this context, integrase gene class 1 in UPL and UDS was distributed in similar percentages among members of *Enterobacteriaceae* (50%), followed by *Pseudomonadaceae*, *Moraxellaceae*, *Alcaligenaceae*, and *Corynebacteriaceae* with values of 10% each. Moreover, quaternary ammonium compound resistance genes were observed in lower proportion of 2.6% in UDS (affiliated only with *Pseudomonadaceae*) compared to 71.6% in UPL. Within this latter watershed, *Enterobacteriaceae* represented 36.8%, followed by *Pseudomonadaceae* and uncultured bacteria with 25% and 23.5%, respectively. We also noted the same pattern of low *qac* in downstream sites compared to sites directly impacted by anthropogenic activities (urban and agricultural). In this context, quaternary ammonium compound resistance genes have been reported from municipal wastewater, hospital, and livestock environments in members of *Pseudomonadaceae* (Wang et al., 2007; Romao et al., 2011; Zou et al., 2014; Wan and Chou, 2015), *Enterobacteriaceae* (Azadpour et al., 2015), *Vibrionaceae* (Kazama et al., 1999) as well as *Enterococcaceae* and *Staphylococcaceae*, indicating this element is present in Gram-negative and Gram-positive bacteria (Kazama et al., 1998).

Members of *Pseudomonadaceae* family were linked to *bla_IMP_* gene in UPL and UDS (**Figure 3B**). Moreover, *tet* genes were found in UPL to be affiliated with the family *Streptomycetaceae* (1.1%). Within the same watershed we also found a chloramphenicol acetyltransferase gene (*catB10*) associated with *Pseudomonadaceae* (1.1%). Members of this family and specifically the genus *Pseudomonas* carrying this gene have been reported in human-impacted environments (Roberts and Schwarz, 2016). Most of ARGs in UDS consisted of *tet* genes (60%) and were distributed in four types of ARGs: *tet(O)* (*Campylobacteriaceae*, 33.3%), *tet(32)*, and *tet(Q)* with 23.8% each, both present in *Clostridiaceae* and *Bacteroidaceae*, respectively, and finally *tet(W)* (*Bifidobacteriaceae*, 19.1%) (**Figure 3B**). *bla*_IMP_ gene was also observed in ADS and associated to *Pseudomonadaceae* (17.1%). More generally, in urban sites we observed determinants of antibiotic resistance such as *intI*, *tet*, and *bla*_IMP_ genes, reported to be associated with wastewater and surface water receiving such effluents (Pellegrini et al., 2009; Zhang et al., 2009; Makowska et al., 2016).

When compared to agricultural and urban locations, a low richness of ARGs (three types) was observed in the protected watershed. In PUP, the *bla*_IMP_ gene represented 60% of ARGs from *Pseudomonadaceae*. While the *bla*_IMP_ gene was also observed in urban sites and agricultural watersheds, significantly different IMP variants (Docquier et al., 2003; Pellegrini et al., 2009) may have been present in each site. We could not type the exact IMP variant based on the recovered sequences. The remaining 40% of ARGs detected in PUP were *bla*_OXA_ genes and distributed evenly between *Pseudomonadaceae* and *Ralstoniaceae* (**Figure 3C**). A class B β-lactamase LRA from uncultured bacteria was the only ARG detected in PDS using metagenomics. In this context, *bla*_LRA_ gene encoding resistance to ampicillin and some members of the cephalosporin structural class such as cefamandole, ceftazidime, and cefoxitin, has been reported in remote Alaskan soil not associated with human activity (Allen et al., 2009). Although richness of ARGs in protected watersheds was low, we hypothesize that the ubiquity of β-lactamases may be related to ancestral/natural β-lactamases present in soil/sediments (Allen et al., 2009; Agga et al., 2015), wildlife (Guenther et al., 2011; Stedt et al., 2015), or biofilms (Schwartz et al., 2003; Balcazar et al., 2015). It is important to note that integrase genes were not detected in any of these protected watershed locations using a metagenomics approach.

Across watersheds, predominant groups of bacteria included *Actinobacteria*, *Firmicutes*, *Bacteroidetes*, and *Proteobacteria* (**Supplementary Spreadsheet File S3**) as previously reported (Peabody et al., 2017). It is important to note that within this latter phyla, *Enterobacteriacea* and *Pseudomonadaceae* represented on average only 1.1% and 2.2% of the total microbial community, respectively. Nevertheless, when combined 39 ARGs and elements of resistance were mostly distributed between *Enterobacteriaceae* (24 gene types) and *Pseudomonadaceae* (26 gene types) families. Moreover, high peaks of relative abundance were observed after T8 in ADS and APL for both families (**Supplementary Figure S3**). APL had higher relative abundance of *Enterobacteriaceae* than AUP (*p* = 0.0424), PUP (*p* = 0.0178), and PDS (*p* < 0.0001), while that for *Pseudomonadaceae* this difference was only detected between APL and PDS (*p*-value = 0.0021). No other significant differences were detected. These fluctuations over time are also reflected in APL and ADS as members of *Pseudomonadaceae* were among the top 50 OTUs observed in these locations (**Supplementary Spreadsheet File S3**). Overall, agriculture-impacted sites were found to have a greater richness of ARGs compared to urban and protected sites. Our metagenomics approach detected *intl1*, a potential indicator of gene cassettes in only the impacted sites, while neither *intI2* nor *intI3* were detected in any of the study sites. Although most of the bacterial community was captured across watersheds as estimated by rarefaction analysis, diversity, and richness indices (**Supplementary Spreadsheet File S3**), detection of functional genes including ARGs by metagenomics is impacted by the relative abundance of other microbes and sequencing depth; whereas the use of quantitative PCR is indifferent to the community structure. Thus, to complement our findings with metagenomics in watersheds further, we used high-throughput qPCR (HT qPCR) analysis.

### Detection and Quantitation of Antibiotic Resistance Genes

To confirm and quantify ARG prevalence in the study watersheds, primers and probes were designed for 60 elements of resistance, based on results from this metagenomics study. Although only class 1 of *intI* genes was detected by metagenomics, we incorporated two other main classes of integrase genes (*intI2* and *intI3*) that have been reported in the literature (Barraud et al., 2010). Because class 1 integron carry *qac* and multiple ARGs (Gaze et al., 2011), *intI1* was also used as indicator of *qac* gene activity.

**FIGURE 4:**
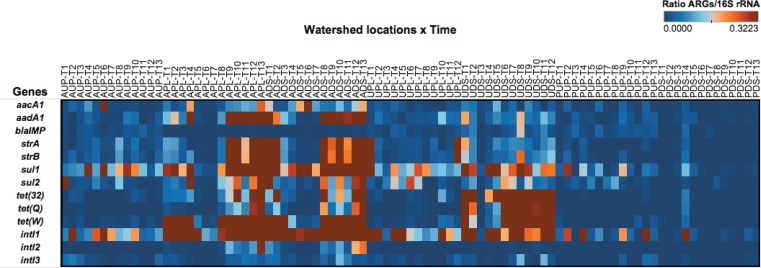
Heat map depicting the ratio of antibiotic resistance genes and 16S rRNA gene over time (as estimated by high-throughput quantitative PCR platform) in watershed sites. AUP, agricultural upstream site; APL, agricultural polluted; ADS, agricultural downstream; UPL, urban polluted; UDS, urban downstream; PUP, protected upstream; PDS, protected downstream. Months are represented by T1 through T13 corresponding to the long-year study (April 2012 to April 2013).

Samples collected from the same sampling locations, but not part of this study, were used to validate qPCR assays for the ARGs. The final HT qPCR panel included a total of 10 ARG, three classes of *intI*, and 16S rRNA gene primers and probes (**Table 1**) which were run on DNA templates from all study samples. The 16S rRNA gene was also included to estimate bacterial counts. A heat map of ARGs normalized by bacterial counts (as estimated by the 16S rRNA gene) shows the relative abundance and distribution of each gene in watershed locations over time (**Figure 4**). Most of the ARGs were estimated to be present in an order of magnitude of 1 × 10^-2^ (**Figure 4**). These findings are in agreement with other studies conducted in agriculture- and urban-influenced environments (Marti et al., 2013; Li B. et al., 2015; Sui et al., 2016). Genes such as *tet(32)*, *tet(Q)*, and *tet(W)* were found in agriculture^-^impacted sites and UDS and had ratios of up to 3.2 × 10^-1^ (relative to the 16S rRNA gene). We hypothesize that these values are related to agricultural discharges and in the UDS site, runoff from storm water could explain the high ratio of tetracycline resistance genes. These gene ratios were also high in agriculture-impacted sites. When ratios were compared across study sites, genes with more distinctive patterns were observed. For instance, genes such as *aacA1*, *aadA1*, *strA*, *strB*, *sul1*, and *sul2* showed high ratios in the agriculture-impacted sites, while relative concentrations as high as 3.2 × 10^-1^ and 1.5 × 10^-1^ were observed for *tet(32)* and *tet(Q)*, respectively in the urban-impacted watersheds. Gene *tet(W)* was prevalent in both urban- and agriculture-impacted sites compared to protected watersheds. We also observed that the *intI1* gene was present over the whole year and in all sites (April 2012 to April 2013). Class 1 integrase genes had ratios ranging from 3.4 to 3.7 × 10^-2^ in ADS and APL, respectively, while those for AUP had a ∼5.8 × 10^-3^ relative concentration. Ratios detected in agriculture-impacted sites were relatively high compared to urban-impacted (1.1–1.9 × 10^-2^) and protected watersheds (1.6–7.7 × 10^-3^). Class 2 integrase genes were detected in agricultural sites (1.6–1.9 × 10^-3^) and to a lesser degree in urban sites ranging from 7.0 × 10^-5^ (UPL) to 1.4 × 10^-4^ (UDS), while in protected sites, *intI2* was only detected once in PUP, at a low relative concentration (8.0 × 10^-5^). Although intermittently observed in the protected watershed locations, integrase class 3 was detected in all watershed locations. On average concentrations of *intI3* were one order of magnitude higher in downstream sites of impacted watersheds (ADS and UDS) compared to the other sites. The prevalence of certain elements of resistance over time may serve as proxies of anthropogenic impacts, as proposed by other studies (Chen et al., 2013; He et al., 2014; Gillings et al., 2015). To elucidate differences over time of ARGs and *intI* in watershed sites, we conducted a longitudinal analysis in terms of sample volume and total extracted bacterial biomass.

### Longitudinal Analysis

**FIGURE 5:**
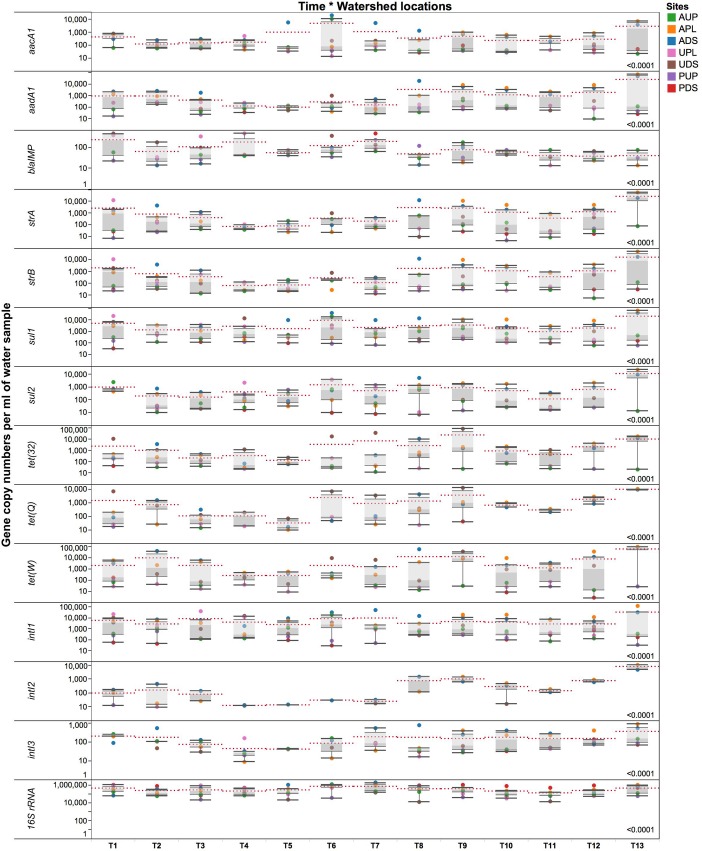
Gene copy numbers of antibiotic resistance genes, integrase gene classes 1, 2, and 3, and 16S rRNA gene per ml of environmental water sample over time in the different locations. AUP, agricultural upstream site; APL, agricultural polluted; ADS, agricultural downstream; UPL, urban polluted; UDS, urban downstream; PUP, protected upstream; PDS, protected downstream. Red dotted lines represent means for a specific time point. Number on the lower right represents *p*-value from the PROC mixed with repeated measures. Statistical significance was set at the 0.05 level.

Absolute numbers generated from standard curves in the HT qPCR platform were normalized per ml of sample (volume) and ng of water DNA (biomass) as described by Ritalahti et al. (2006). Due to the multicopy nature of the 16S rRNA gene, a factor of 4.3 was used to normalize bacterial counts (Lee et al., 2009). **Figure 5** and **Supplementary Figure S4** depict GCNs per ml of water sample and ng DNA, respectively, for all watershed study sites. Longitudinal analysis revealed striking differences between watershed sites in terms of 16S rRNA gene, ARGs, and *intI* genes. Copy numbers of the 16S rRNA gene in orders of magnitude of 10^5^ (volume) were detected and found to change over time within the watersheds. When comparing sites, we observed average quantities of the 16S rRNA gene with 7.34 × 10^5^ GCN/ml sample in PDS, followed by ADS and APL locations with 5.67 × 10^5^ and 4.17 × 10^5^ GCN/ml of sample, respectively. It is probable that values observed in agriculture impacted watersheds are associated with farm discharges. We propose that the high GCN in PDS may be due to biofilm in the pipe where study samples were collected (Uyaguari-Diaz et al., 2016) as noted above.

In the agriculture-impacted sites, significantly higher GCNs of *aacA1*, *aadA1*, *strA*, *strB*, *sul1*, *sul2*, *intI* classes 1–3 were detected over time compared to the protected watersheds (**Figure 5**). Genes such as *tet(32)*, *tet(Q)*, and *tet(W)* were more abundant in both the agriculture- and urban-impacted sites compared to the protected and upstream sites. During this yearlong study, integrase classes 1, 2, and 3 appeared to be predominant in APL and ADS. Moreover, the means of *intI* genes differed (*p* < 0.0001) over time per watershed location. In agricultural sites, *intI* class 1, 2, and 3 genes were detected in at least one order of magnitude higher than in urban or protected watersheds, with values ranging from 10^4^ to 10^5^, 10^2^ to 10^4^, and 10^2^ to 10^3^ GCNs/ml of sample for *intI1*, *2*, and *3*, respectively. Integrase class 1 genes were detected in all sites; GCNs were lower in the protected watersheds compared to the agriculture- and urban-impacted watersheds. Integrase class 2 was mostly observed in APL and ADS over time, while *intI3* was detected in higher GCNs in agricultural and urban sites. It was observed that in some sampling events *intI2* (T1) or *intI3* (T10–T12) were detected in PDS, but in significantly lower GCNs compared to the impacted sites. We also observed that most of these significant changes in absolute GCNs per ml of water occurred around October (T7), which coincides with the beginning of the rainy season and subsequently, greater microbial transport. For instance, higher GCNs per ml of water (after T7) were detected for genes such as *aadA1*, *strA*, *strB*, *tet(32)*, *tet(Q)*, *tet(W)*, *intI2*, and *intI3* (**Figure 5**). Although patterns and predictions of ARGs may differ among aquatic systems and gene types, significant increases in absolute GCNs of ARGs have been reported associated to rainfall and seasonality (Di Cesare et al., 2015, 2017). On the contrary and within this context, GCNs of *bla*_IMP_, were higher in urban-impacted watersheds from T1 to T7 (**Figure 5**); followed by the onset of the rainy season, a slight decline in GCNs of *bla*_IMP_ was detected and persisted over time. Although AUP is not directly impacted by agriculture activities, the presence of some residences near the sampling site may have some influence in the *bla*_IMP_ gene patterns observed.

An analysis of GCNs expressed per ng DNA (**Supplementary Figure S4**) revealed seasonal downstream transport of ARGs in the sampling sites. We observed that these intermittent changes occurred at different times of the year. From T1 through T8 (April to November 2012), high GCNs of *aacA1*, *sul1*, *sul2*, *intI1*, and *intI2* were detected in ADS compared to APL. During the same time period (T1–T8), high GCNs per ng DNA of *bla_IMP_*, *tet(32)*, and *tet(Q)* were observed in UDS compared to UPL (**Supplementary Figure S4**). Moreover, a downstream transport of genes such as *aadA1*, *strA*, *strB*, and *tet(W)* was observed in both agricultural and urban downstream locations. Low GCNs of ARGs were detected during T4 and T5 in all watershed locations (**Figure 5** and **Supplementary Figure S4**), the driest months (July and August) in southwestern British Columbia. This pattern of seasonal variation was similar for most ARGs described (**Supplementary Figure S4**). After T8 and with the onset of the rainy season, higher GCNs were observed. No transport patterns, however, were observed downstream from the impacted sites. Seasonal changes have been documented in other freshwater ecosystems studies (Knapp et al., 2012). Overall, some gene patterns in terms of seasonality were observed between the dry (time points 1–7, corresponding to spring and summer) and rainy season (time points 8–13, corresponding to fall and winter; **Figure 5**); more time series studies across seasons are needed to confirm this observation.

### Exploratory Factor Analysis of Watershed Microbiomes

**FIGURE 6:**
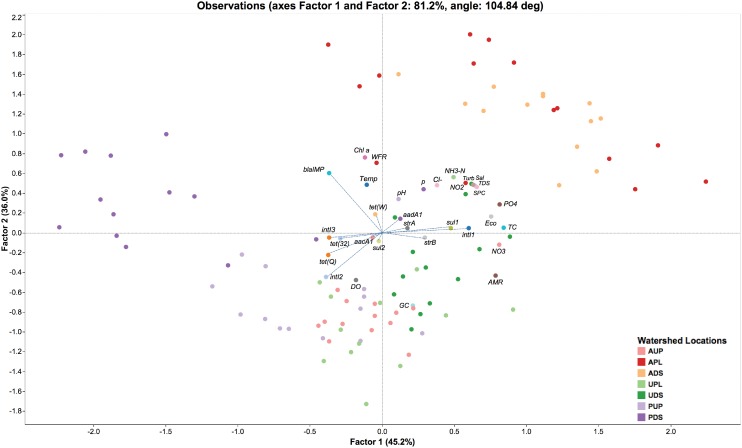
Factor analysis of antibiotic resistance genes/16S rRNA gene and environmental variables observed over time in watershed locations. AUP, agricultural upstream site; APL, agricultural polluted; ADS, agricultural downstream; UPL, urban polluted; UDS, urban downstream; PUP, protected upstream; PDS, protected downstream. Factor 1 and Factor 2 represent environmental stressors and aerobic conditions, respectively. AMR, percentage of antibiotic resistance genes found in metagenomic sequences (based on CARD and Integrall); Chl a, chlorophyll a; Cl^-^, dissolved chloride; DO, dissolved oxygen; Eco, *E. coli* counts; GC, percentage of guanine-cytosine content; NH_3_-N, ammonia; NO_2_, nitrite; NO_3_, nitrate; pH, potential of hydrogen; PO_4_, orthophosphate; Sal, salinity; SPC, specific conductivity; TC, total coliform counts; TDS, total dissolved solids; Temp, temperature; Turb, turbidity; WFR, water flow rate. Blue dashed lines represent factor loading values for antibiotic resistance genes and integron-integrase genes.

An exploratory factor analysis was conducted using the ratios of ARGs to the 16S rRNA gene with physicochemical and biological parameters from each study watershed. Both orthogonal and oblique rotations were conducted. **Figure 6** depicts an oblique parsimax rotation that best fits all variables assessed in this study. Factor 1 or “anthropogenic stressors” accounted for 45.2% of the observed variability, while that factor 2 or “aerobic conditions” accounted for 36.0% of the observed variability. Four clusters can visually be identified (**Figure 6**): APL and ADS; AUP and UPL; UDS; PUP; and PDS. Integrase genes class 1, *sul1*, *sul2*, *strA*, *strB*, *aacA1*, *aadA1*, and *tet(W)* appear to be driven by anthropogenic stressors in agriculture impacted sites (APL and ADS), UDS, and to a lesser extent UPL. Moreover, nutrients associated with land-use such as orthophosphate, nitrate, nitrite, and ammonia (Fraterrigo and Downing, 2008; Barnes and Raymond, 2010; Soranno et al., 2015) were observed within the same quadrant as these ARGs. In additional models, where metadata was excluded from the analysis or where only nutrients were incorporated into the model, *intI1* and *sul1* clustered within the same quadrant as anthropogenic perturbations (data not shown). Furthermore, a significant positive correlation (*r* = 0.8078, *p* < 0.0001) was detected between these two genes (**Supplementary Figure S5**). This finding is in agreement with previous studies linking *intI1* and *sul1* to anthropogenic activities in freshwater ecosystems (Pruden et al., 2012; Wang F.H. et al., 2014; Gillings et al., 2015). Note that loading of traditional markers of water quality (total coliform and *E. coli*) also fell within the same quadrant as agricultural impacted sites. While the presence of these indicator organisms does not necessarily indicate the presence of harmful bacteria in water, their counts in APL and ADS may be heavily associated with farm discharges, effluents from human septic systems, as well as from non-point source fecal contamination such as wildlife or human recreational activities (Harmel et al., 2010; Walters et al., 2011).

On the other hand, ratios of *tet(Q)* appeared to be related to aerobic conditions as observed in AUP, UPL, and PUP (**Figure 6**). A significant positive correlation (*p* < 0.0001) were detected between this gene and dissolved oxygen (DO). *tet(Q)* has primarily been associated with anaerobic bacteria, and in a lesser extend to aerobic and facultative anaerobic bacteria (Chung et al., 1999; Roberts and Schwarz, 2009, 2016). It is possible that the latter groups of bacteria carrying *tet(Q)* may have driven the relative abundance of this gene in these watersheds. Whereas *intI2* was positively correlated with DO (*r* = 0.4214, *p* < 0.0001) but rarely detected in non-impacted sites (**Figure 5**), we hypothesize that their relative abundance in agricultural watersheds aligns with the presence of aerobic bacterial hosts of integron genes rather than anthropogenic stressors or other environmental factors. Furthermore, integrase class 2 and *tet(Q)* were positively correlated among them (*r* = 0.5450, *p* < 0.0001). It is possible that aerobic conditions favored the ratio of these genes compared to the overall genes in a microbial community.

When comparing ratios of *tet(32)* and *intI3*, they were not affected by environmental stressors or aerobic conditions (**Supplementary Figures S6**, **S7**). Instead, natural factors may influence their occurrence in the studied watersheds. For instance, *tet(32)*, previously documented in only anaerobic bacteria from clinical samples (Melville et al., 2001; Warburton et al., 2009) has recently been reported to be widespread in human, animal, and environmental resistomes (Pal et al., 2016). Another example is the wide distribution of the lesser known integrase gene, *intI3*, in natural environments (Barkovskii et al., 2010; Uyaguari et al., 2013). It is important to mention that biofilms in PDS (as noted above) may have also had an effect on the microbial community structure and thus of the naturally occurring ARGs. The role of biofilms as reservoirs of ARGs has been well documented as has their influence on increasing resistance of bacteria to antibiotics (10–1000 times compared to free-living bacteria; Gilbert et al., 2002; Sabater et al., 2007; Balcazar et al., 2015). Finally, the ratio of *bla*_IMP_ seems as well to be aligned with the natural occurrence of bacteria in watersheds. This metallo-β-lactamase gene conferring resistance to carbapenems was positively correlated (*p* ≤ 0.0233) with parameters such chlorophyll *a* and water flow rate (**Figure 6** and **Supplementary Figures S5**–**S7**). Although an inverse relationship between chlorophyll *a* and water flow rate has been widely reported in freshwater ecosystems (Reynolds, 2000; Salmaso and Braioni, 2008; Lucas et al., 2009; Li et al., 2010; Desortova and Puncochar, 2011), this condition only applied in the more impacted watersheds (APL, ADS, UPL, and UDS). In this study, we observed a positive correlation between both parameters in lesser-impacted environments such as AUP or non-impacted watersheds such as PUP. No association could be determined in PDS, perhaps due to the influence of pipe-associated biofilms on this site. Another correlation (*p* = 0.0001) was observed between temperature and chlorophyll a, but this factor did not seem to have a major effect (*p* = 0.2733) on the population density harboring the *bla*_IMP_ gene. It should be noted that absolute GCNs of *bla*_IMP_ in urban, AUP, and protected watersheds were detected in at least one order of magnitude higher than APL or ADS (**Figure 5** and **Supplementary Figure S4**). Additional analysis of phylogenetic information associated to *bla*_IMP_ genes suggested that species of *Pseudomonas* (**Figure 3**) are the most probable bacterial host for this gene. Members of this genus are important phytopathogens and are also opportunistic agents of human infections (Tripathy et al., 2007). They have been reported in low nutrient or oligotrophic environments, urban environments, colonizing biofilms, and plumbing structures (Mena and Gerba, 2009). These observations may explain the relative abundance of *bla*_IMP_ in non-agricultural impacted sites.

The model described in this study included only three of the major factors that may explain the overall variability of ARGs in watershed locations. Besides land-use practices, additional factors may have influenced the pattern of bacterial communities and ARGs in watersheds, such as seasonal conditions, water flow (i.e., flow in ADS is regulated by gated dams located 8.7 km further downstream), and indirect human interventions (i.e., water collected in PDS passes through a pipe) (Knapp et al., 2012; Zhang et al., 2015).

### Analysis of Antibiotic Residues in Freshwater Samples

To further understand the impact of antibiotics on the aquatic environment, we screened for antibiotic residues using a subset of water samples. The selection of antibiotic metabolites screened was derived from the metagenomics analysis supported with information on the most commonly employed antibiotics for agricultural and human purposes in Canada (Government of Canada, 2015, 2016; Agunos et al., 2016). Information from antibiotics used on farms was not available for this study area (Henrich et al., 2015) compared to other studies (Li et al., 2013; Zhu et al., 2013; Zhao et al., 2016). Detection limits for the analytical methods used were relatively low for each antibiotic as follows: ampicillin (0.020 μg/l), sulfamethoxazole (0.0050 μg/l), chlortetracycline (0.025 μg/l), doxycycline (0.050 μg/l), oxytetracycline (0.010 μg/l), and tetracycline (0.025 μg/l). On analysis, none of the three groups of antibiotics were detectable in the subset of water samples and as such, further testing on the entire dataset (*n* = 89) was discontinued. For various reasons, it is probable that the amounts of antimicrobials used in this part of Canada are a smaller fraction of those used in other countries (i.e. China; Yin et al., 2013; Mao et al., 2015). Other factors such as a rapid degradation (including environmentally relevant conditions; Soeborg et al., 2004; Sanderson et al., 2005; Zhang et al., 2013; Li et al., 2016), formation of metabolites (even at a higher concentration than the parent molecule; Halling-Sorensen et al., 2002; Kolpin et al., 2002; Bonvin et al., 2013; Jiang et al., 2013), and distribution (into sediments rather than the water column; Li et al., 2012; Liang et al., 2013; Chen and Zhou, 2014; Xu et al., 2014) may have resulted in these antibiotics being below the detection limit. On the other hand, it is known that low concentrations of antibiotics or their metabolites have been associated with selectivity for antibiotic resistant bacteria (Gullberg et al., 2011; Sandegren, 2014). The observation of high richness of ARGs in the agriculture-impacted sites compared to the urban (∼1:7 ratio) and protected (∼1:11 ratio) sites suggests that the ARGs were more likely derived from bacteria from the effluents rather than the *de novo* acquisition of resistance in the naturally occurring bacteria in the water. Finding greater GCNs of ARGs quantified in agriculture and urban impacted sites compared to non-impacted watershed locations also supports this.

## Conclusion

The total number of sequences associated with ARGs from bacterial reads were low in aquatic environments (<1%). The metagenomics approach identified a total of 46 different ARGs, one integrase class type 1, a groEL/*intI1* gene, and 12 *qac* genes across all sampling sites. Agriculture-impacted sites contained a higher richness of ARGs compared to the urban or protected environments. Thirteen genes using HT qPCR were further screened to quantify GCNs of ARG in the study sites. We included integrase gene classes 1, 2, and 3 due to their relevance to mobile genetic elements and ARGs. We detected higher GCNs of ARGs in agriculture and urban impacted sites than in protected sites. A downstream transport pattern was identified for most of the ARGs during the dry season, while these differences became undetectable with the onset of higher precipitation in the study area. Genes such as *aacA1*, *aadA1*, *strA*, *strB*, *sul1*, *sul2*, and *intI2* were more prevalent in agricultural sites, while *tet(32)* and *tet(Q)* had a higher prevalence in the urban sites. Genes *tet(W)* and *intI1* were prevalent in both urban and agricultural settings. Moreover, an exploratory factor analysis found that there were three major contributors/drivers of ARGs in the watershed study sites: anthropogenic stressors (45.2%), aerobic conditions (36.0%), as well as natural occurrence (18.8%). The inability to detect antibiotics in the water suggests that the ARGs may have come from organisms in effluents from impacted sites. This is consistent with the resilience/stability of antibiotic resistant organisms even after they enter the environment. Although the occurrence of ARGs in these sites was low compared to the bacterial population, high richness and GCNs in agricultural sites and to a lesser extent in urban sites demonstrates the influence of anthropogenic activities on the aquatic environment.

## Author Contributions

MU-D conceived, designed, and performed the experiments and wrote the manuscript. MaC led the bioinformatic analyses and designed the experiments. ZL designed and performed the experiments. KC, MiC, SL, and WB performed the experiments. MN performed the size selection of sequencing libraries. DD and WH provided additional bioinformatic analyses. MU-D, MaC, KM, JI-R, PT, and NP designed the experiments, contributed analysis tools, guided the analyses, and aided in interpretations. All authors contributed to final revisions of the manuscript.

## Conflict of Interest Statement

MN holds shares of Coastal Genomics, a privately owned British Columbia company offering the Ranger Technology used in this study. The other authors declare that the research was conducted in the absence of any commercial or financial relationships that could be construed as a potential conflict of interest.

## Footnotes

1 http://www.ncbi.nlm.nih.gov/bioproject/287840

## Supplementary Material

FIGURE S1 Sampling locations with land cover: **(A)** Agricultural watershed, **(B)** Urban watershed, and **(C)** Protected watershed.

FIGURE S2 Pie chart depicting the relative abundance of antibiotic resistance gene categories identified by CARD in watershed locations: **(A)** Group of antibiotic, and **(B)** Mechanism of action. This figure includes all contigs identified by CARD that were assigned as housekeeping function-associated genes (i.e., elongation factors, DNA-directed RNA polymerase and DNA topoisomerases/gyrases, resistance nodulation cell division, and transport systems).

FIGURE S3 Line plots of *Enterobacteriaceae* and *Pseudomonadaceae* observed in watershed locations. AUP: agricultural upstream site; APL: agricultural polluted; ADS: agricultural downstream; UPL: urban polluted; UDS: urban downstream; PUP: protected upstream; PDS: protected downstream. The *x*-axis represents sample collection time (T1–T13) and the *y*-axis shows percentages of relative abundance compared to the microbial community using shotgun metagenomics.

FIGURE S4 Gene copy numbers of antibiotic resistance genes, integrase gene classes 1, 2, and 3, and 16S rRNA gene per ng of DNA over time in watershed locations. AUP, agricultural upstream site; APL, agricultural polluted; ADS, agricultural downstream; UPL, urban polluted; UDS, urban downstream; PUP, protected upstream; PDS, protected downstream. Red dotted lines represent mean values for a specific time point. Number on the lower right represents *p*-value from the PROC mixed with repeated measures. Statistical significance was set at the 0.05 level.

FIGURE S5 Heat map showing the Spearman’s rank correlation analysis between integrase and antibiotic resistance genes and water quality parameters.

FIGURE S6 Factor analysis of antibiotic resistance genes/16S rRNA gene and environmental variables observed over time in watershed locations. AUP, agricultural upstream site; APL, agricultural polluted; ADS, agricultural downstream; UPL, urban polluted; UDS, urban downstream; PUP, protected upstream; PDS, protected downstream. Factor 1 and Factor 3 represent environmental stressors and natural occurrence, respectively. AMR, percentage of antibiotic resistance genes found in metagenomic sequences (based on CARD); Chl a, chlorophyll a; Cl^-^, dissolved chloride; DO, dissolved oxygen; Eco, *E. coli* counts; GC, percentage of guanine-cytosine content; NH_3_-N, ammonia; NO_2_, nitrite; NO_3_, nitrate; pH, potential of hydrogen; PO_4_, orthophosphate; Sal, salinity; SPC, specific conductivity; TC, total coliform counts; TDS, total dissolved solids; Temp, temperature; Turb, turbidity; WFR, water flow rate. Blue dashed lines represent factor loading values for antibiotic resistance genes and integron-integrase genes.

FIGURE S7 Factor analysis of antibiotic resistance genes/16S rRNA gene and environmental variables observed over time in watershed locations. AUP, agricultural upstream site; APL, agricultural polluted; ADS, agricultural downstream; UPL, urban polluted; UDS, urban downstream; PUP, protected upstream; PDS, protected downstream. Factor 2 and Factor 3 represent aerobic conditions and natural occurrence, respectively. AMR: percentage of antibiotic resistance genes found in metagenomic sequences (based on CARD); Chl a: chlorophyll a; Cl^-^: dissolved chloride; DO: dissolved oxygen; Eco: *E. coli* counts; GC: percentage of guanine-cytosine content; NH_3_-N: ammonia; NO_2_: nitrite; NO_3_: nitrate; pH: potential of hydrogen; PO_4_: orthophosphate; Sal: salinity; SPC: specific conductivity; TC: total coliform counts; TDS: total dissolved solids; Temp: temperature; Turb: turbidity; WFR: water flow rate. Blue dashed lines represent factor loading values for antibiotic resistance genes and integron-integrase genes.

SPREADSHEET FILE S1 Summary of Primer pair collision detection.

SPREADSHEET FILE S2 Number of contigs matching CARD and Integrall.

SPREADSHEET FILE S3 Microbial community analysis.
