# Supplementary figures and images for: Human Activity Determines the Presence of Integron-Associated and Antibiotic Resistance Genes in Southwestern British Columbia
Source: Front Microbiol. 2018 May 1;9:852. doi: 10.3389/fmicb.2018.00852 (PMC5938356; doi:10.3389/fmicb.2018.00852)

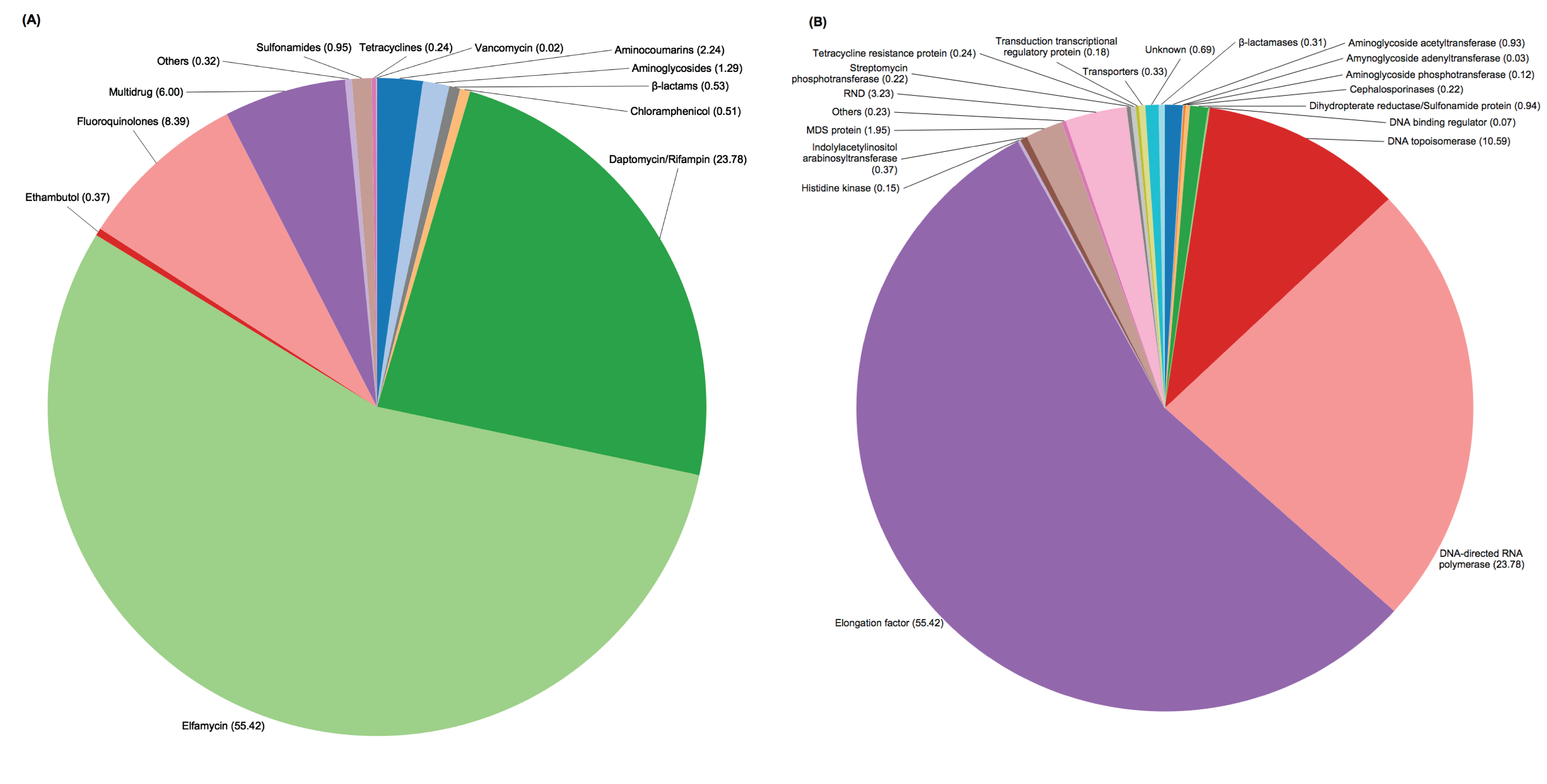

Supplement: FIGURE S2 — Pie chart depicting the relative abundance of antibiotic resistance gene categories identified by CARD in watershed locations: (A) Group of antibiotic, and (B) Mechanism of action. This figure includes all contigs identified by CARD that were assigned as housekeeping function-associated genes (i.e., elongation factors, DNA-directed RNA polymerase and DNA topoisomerases/gyrases, resistance nodulation cell division, and transport systems). [file Image_2.TIFF]

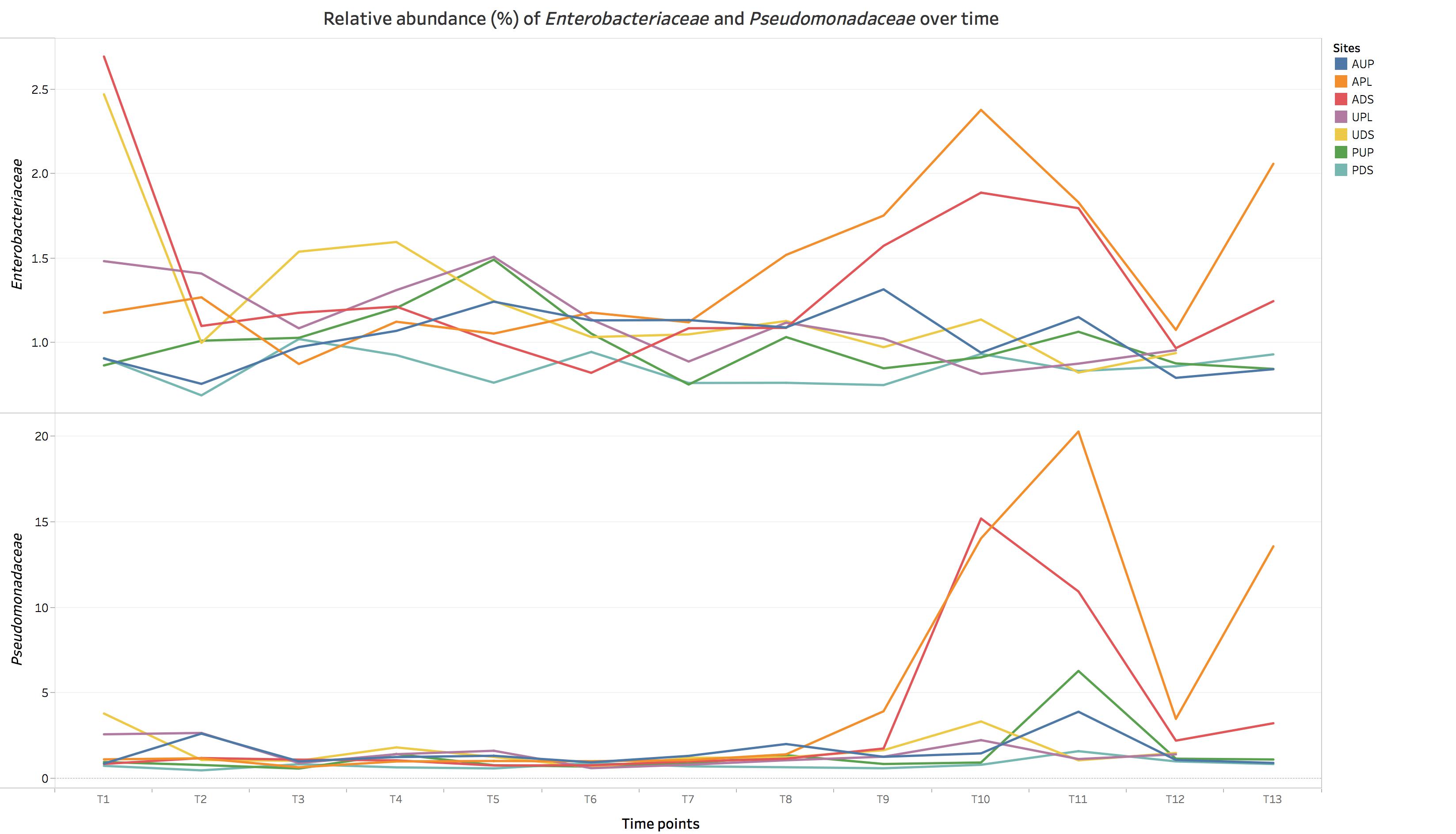

Supplement: FIGURE S3 — Line plots of Enterobacteriaceae and Pseudomonadaceae observed in watershed locations. AUP: agricultural upstream site; APL: agricultural polluted; ADS: agricultural downstream; UPL: urban polluted; UDS: urban downstream; PUP: protected upstream; PDS: protected downstream. The x-axis represents sample collection time (T1–T13) and the y-axis shows percentages of relative abundance compared to the microbial community using shotgun metagenomics. [file Image_3.JPEG]

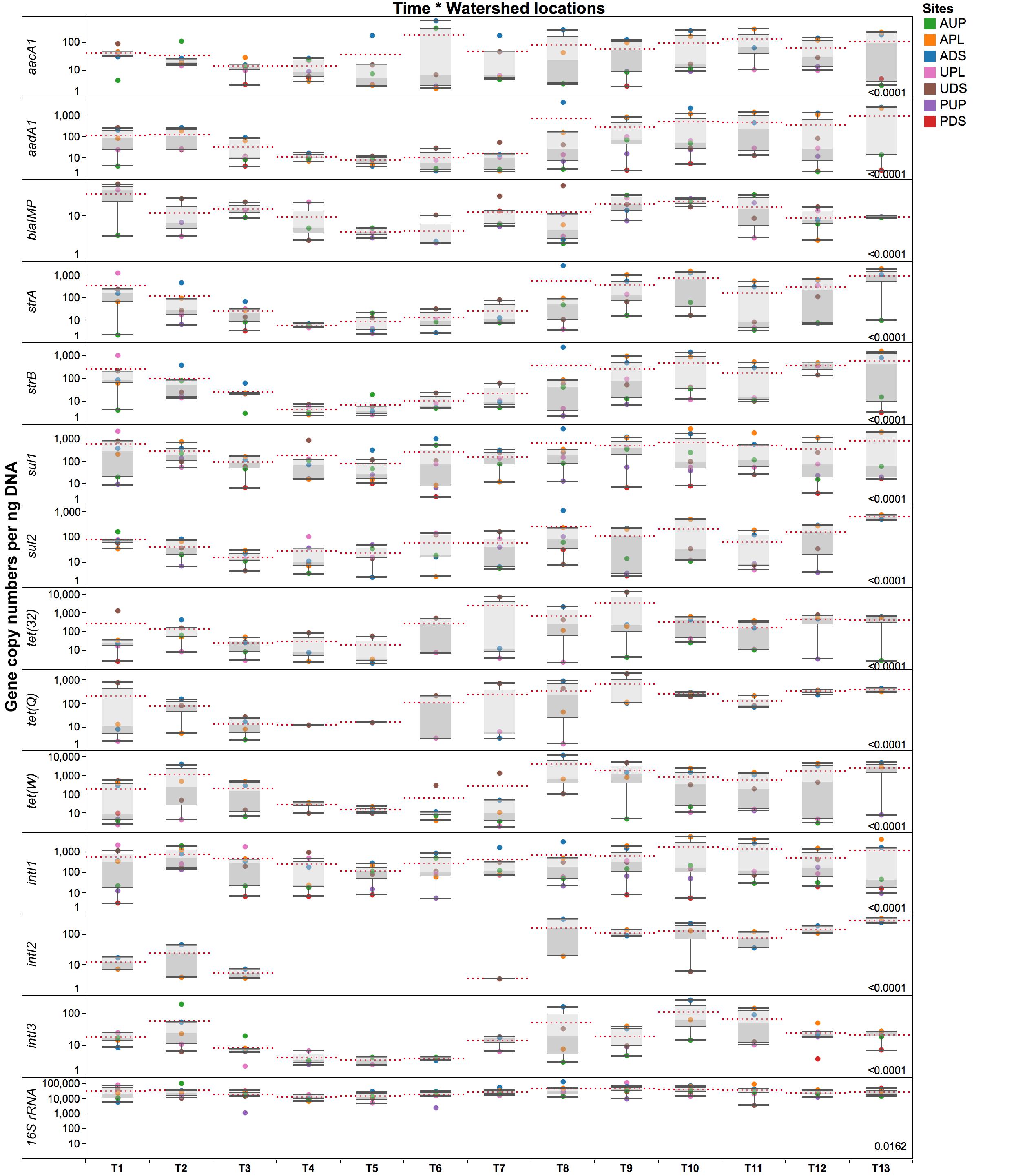

Supplement: FIGURE S4 — Gene copy numbers of antibiotic resistance genes, integrase gene classes 1, 2, and 3, and 16S rRNA gene per ng of DNA over time in watershed locations. AUP, agricultural upstream site; APL, agricultural polluted; ADS, agricultural downstream; UPL, urban polluted; UDS, urban downstream; PUP, protected upstream; PDS, protected downstream. Red dotted lines represent mean values for a specific time point. Number on the lower right represents p-value from the PROC mixed with repeated measures. Statistical significance was set at the 0.05 level. [file Image_4.JPEG]

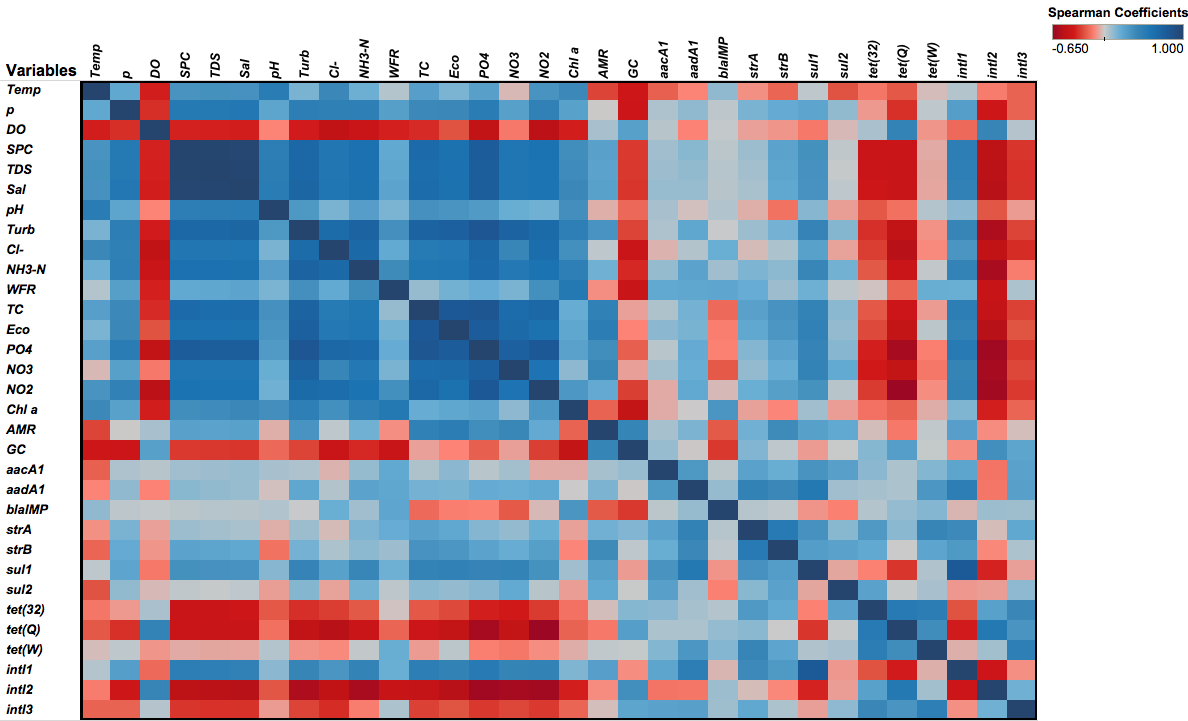

Supplement: FIGURE S5 — Heat map showing the Spearman’s rank correlation analysis between integrase and antibiotic resistance genes and water quality parameters. [file Image_5.TIFF]

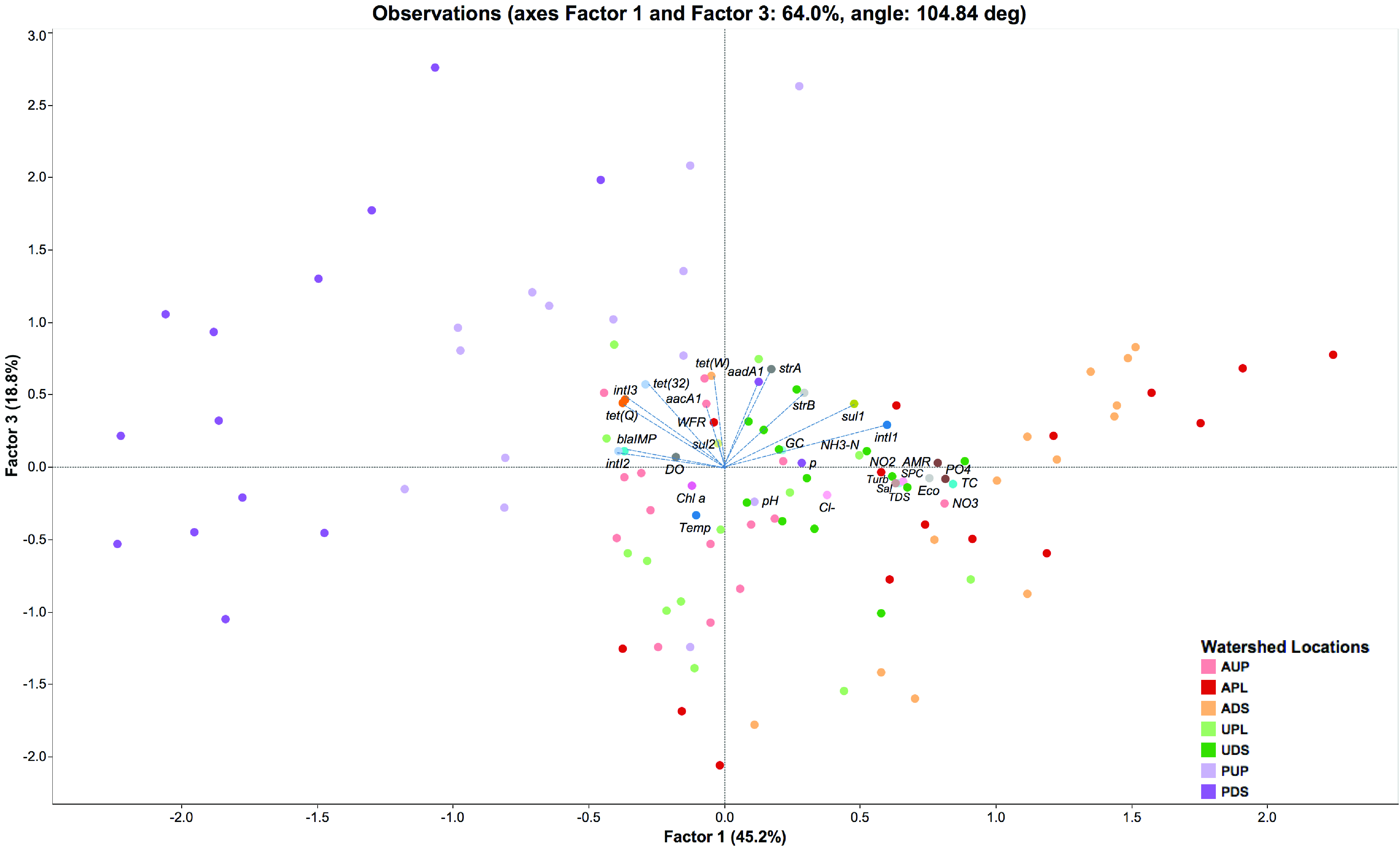

Supplement: FIGURE S6 — Factor analysis of antibiotic resistance genes/16S rRNA gene and environmental variables observed over time in watershed locations. AUP, agricultural upstream site; APL, agricultural polluted; ADS, agricultural downstream; UPL, urban polluted; UDS, urban downstream; PUP, protected upstream; PDS, protected downstream. Factor 1 and Factor 3 represent environmental stressors and natural occurrence, respectively. AMR, percentage of antibiotic resistance genes found in metagenomic sequences (based on CARD); Chl a, chlorophyll a; Cl-, dissolved chloride; DO, dissolved oxygen; Eco, E. coli counts; GC, percentage of guanine-cytosine content; NH3-N, ammonia; NO2, nitrite; NO3, nitrate; pH, potential of hydrogen; PO4, orthophosphate; Sal, salinity; SPC, specific conductivity; TC, total coliform counts; TDS, total dissolved solids; Temp, temperature; Turb, turbidity; WFR, water flow rate. Blue dashed lines represent factor loading values for antibiotic resistance genes and integron-integrase genes. [file Image_6.TIF]
